# Intensification of Treosulfan-Fludarabine Conditioning With Thiotepa Exhibited Effectiveness and Tolerability in Older or Comorbid Patients Undergoing Allogeneic Hematopoietic Stem Cell Transplant With Active Myeloid Neoplasm: A Real-world Study

**DOI:** 10.1097/TXD.0000000000001896

**Published:** 2026-01-21

**Authors:** Luca Tosoni, Gabriele Facchin, Rosa Plos, Chiara Callegari, Matteo Fanin, Maria De Martino, Marta Lisa Battista, Antonella Geromin, Renato Fanin, Francesca Patriarca

**Affiliations:** 1 Division of Hematology and Stem Cell Transplantation, University Hospital ASUFC, Udine, Italy; 2 Department of Medicine (DMED), University of Udine, Udine, Italy

## Abstract

**Background.:**

Reduced-intensity regimens such as treosulfan-fludarabine (TF) are currently considered cornerstones for older or comorbid patients with acute myeloid leukemia and myelodysplastic syndrome (AML/MDS) undergoing allogeneic hematopoietic stem cell transplant (alloSCT). However, data on its use in active disease cases are limited, and intensification strategies (such as thiotepa addition [TTF]) are not standardized in this setting.

**Methods.:**

To evaluate the efficacy and tolerability of treosulfan-based conditioning in the real-world setting and to provide insights into the use of thiotepa intensification, we retrospectively analyzed 66 (AML, n = 58; MDS, n = 8) patients transplanted between January 2016 and December 2023 at the Hematology Division of Udine, receiving TF (n = 48) or TTF (n = 18).

**Results.:**

At alloSCT, the median age was 67 y; 37 patients (56%) had a hematopoietic cell transplantation-comorbidity index of >2, and 13 (20%) had received previous alloSCT. Complete remission status was achieved by 52 (79%). A matched donor was used in 35 cases (53%). With a median follow-up of 16 mo, the cumulative incidences of 100-d grade ≥2 acute graft-versus-host disease and 3-y moderate-to-severe chronic graft-versus-host disease were 34% and 21%, respectively. After 3 y, cumulative incidences of nonrelapse mortality and disease relapse were 18% and 40%, with 46% progression-free survival and 52% overall survival. The TTF cohort included more cases with active disease status at alloSCT, both cytological (43% versus 12%, *P *= 0.005) and minimal residual disease positive (90% versus 40%, *P *= 0.005), compared with TF. Nevertheless, survival analysis confirmed no difference in nonrelapse mortality, progression-free survival, and overall survival among groups.

**Conclusions.:**

Our results confirmed the efficacy and tolerability of the TF regimen in older or comorbid AML and MDS patients in the real-world setting and suggest that double alkylation might counterbalance the adverse outcome risk posed by active disease at alloSCT. Furthermore, larger, prospective studies are needed to better elucidate the patients for whom the addition of thiotepa might be most beneficial.

## INTRODUCTION

Allogeneic hematopoietic stem cell transplant (alloSCT) represents the only potentially curative option for many adults affected by acute myeloid leukemia (AML) and myelodysplastic syndrome (MDS); however, the choice of conditioning regimen can be challenging for older or comorbid patients. In this setting, treosulfan-fludarabine (TF) showed a potent antileukemic effect with modest organ toxicity on the basis of some phase II studies.^1,2^ A randomized phase III trial demonstrated that TF conditioning improved outcomes in older and unfit patients with AML and MDS undergoing alloSCT compared with a reduced intensity busulfan-fludarabine regimen.^3^ Treosulfan advantage was consistently observed in patients aged between 50 and 70 y and/or with a comorbidity index (hematopoietic cell transplantation-comorbidity index [HCT-CI]) ≥2, with AML in complete remission (CR) or MDS with <20% marrow blasts, receiving HLA-identical or matched unrelated donor grafts.^4^ A more recent comparative European Bone Marrow Transplantation (EBMT) registry study on this setting revealed TF superiority against fludarabine-melphalan and busulfan-cyclophosphamide through a matched pair analysis.^5^ Notably, treosulfan-based regimen showed reduced severe acute graft-versus-host disease (GVHD) rates and related deaths, compared with the thiotepa-busulfan-fludarabine regimen in the haploidentical donor AML transplant setting.^6^ Based on these results, TF emerged as the new standard reduced-intensity conditioning regimen in older or comorbid AML and MDS patients. All these findings were achieved using treosulfan in patients undergoing alloSCT for myeloid neoplasms in CR. Thiotepa added to busulfan and fludarabine has proven to be an effective preparative regimen before haploidentical donor and cord blood transplants or matched sibling and unrelated donor transplants, demonstrating excellent antileukemic activity and limited toxicity, even when combined with high-dose busulfan.^7,8^ Furthermore, a few recent studies investigated the efficacy and safety of thiotepa in combination with TF (TTF) in AML and MDS, highlighting high feasibility and antileukemic effect in patients undergoing first and second alloSCT.^9,10^

However, there are no large data on the direct comparison of TF and TTF. Hence, we report the experience of our single-center cohort of AML and MDS patients who received TTF or TF regimens before alloSCT. The primary objective of this study was to describe the tolerability of TF and TTF regimens, estimate nonrelapse mortality (NRM), organ toxicity and infection rates, and assess the effectiveness of progression-free survival (PFS) and overall survival (OS). The secondary objective was to investigate engraftment and immune reconstitution in the 2 groups.

## MATERIALS AND METHODS

### Study Population

This study is a single-center, retrospective analysis of consecutive patients who received TF or TTF conditioning before alloSCT for AML or MDS at the Hematology Division of Udine from January 2016 to December 2023. A total of 66 adult patients were included in total. On admission to the transplant center, all patients signed a consent form in which they expressed their consent to medical data collection for the EBMT registry in an anonymous form and to its sharing for clinical study purposes. All data of this study are based on this registry. Approval for the use of the consent form was granted by the local Ethics Committee (protocol No. 004632/P/GEN/ARCS). Patient comorbidity burden at transplant was assessed via the HCT-CI score.^11^ Disease diagnosis and disease risk index were assessed in accordance with the most recent World Health Organization classification in effect at the time.

### Conditioning Regimen and GVHD Prophylaxis

TF combination was the regimen of choice in adult patients with at least 65 y or ineligible for a myeloablative regimen due to the comorbidity burden (HCT-CI >2) or history of previous alloSCT. All patients received treosulfan 10 g/m^2^ dose per 3 d plus fludarabine 30 mg/m^2^ per 5 d. Intensification with the addition of thiotepa 5 mg/kg to TF was performed in patients with active disease at alloSCT (marrow blasts between 5% and 20% on cytological examination). However, 6 patients with active disease received TF instead of TTF due to previous alloSCT history and high comorbidity burden (HCT-CI >2). Conditioning regimen intensity was low in all patients in accordance with the transplant conditioning intensity score.^12^ Graft source was either peripheral blood or bone marrow. Posttransplant cyclophosphamide (PTCy), mycophenolate mofetil, and calcineurin inhibitor (cyclosporine or tacrolimus) combination was used as GVHD prophylaxis for haploidentical donors, whereas antithymocyte globulin (ATG), calcineurin inhibitor, and methotrexate were administered in matched related and unrelated donors. Mismatched unrelated donor transplants received ATG-based GVHD prophylaxis until 2018, whereas PTCy-based one was preferred from 2018 onward. Immunosuppression prophylaxis reduction started between 3 and 4 mo after alloSCT and was stopped between 6 and 9 mo after alloSCT based on disease relapse risk, drug toxicity, and GVHD status.

### Supportive Care

All patients received antiviral, Pneumocystis jurovecii pneumonia, antibacterial, and antifungal prophylaxis. Acyclovir and trimethoprim-sulfamethoxazole were continued until 12 mo after alloSCT. Levofloxacin was continued until neutrophil engraftment. Posaconazole was preferred over fluconazole in unmatched transplants or in the presence of active disease. Caspofungin and liposomal amphotericin B were considered in case of posaconazole contraindications or toxicity. Cytomegalovirus-seropositive recipients were all given letermovir prophylaxis. All patients received ursodeoxycholic acid as veno-occlusive disease (VOD) prophylaxis.

### Disease Monitoring, Engraftment, and Measures of Immune Reconstitution

Bone marrow and peripheral blood samples were collected at 1, 3, 6, and 12 mo after alloSCT for disease monitoring, chimerism assessment, and lymphocyte subsets analysis. Minimal residual disease (MRD) status was assessed by polymerase chain reaction analysis if a molecular marker was available. Flow cytometry (BD FACSCanto II) was used to define MRD status in the absence of a molecular marker and to evaluate immune reconstitution.^13^ Neutrophil and platelet engraftment were defined as an absolute neutrophil count of ≥0.5 × 10^9^/L for 3 consecutive days and platelet count ≥20 × 10^9^/L, unsupported by transfusions for 7 d, respectively. Full-donor chimerism was assessed through microsatellite analysis (AB 3500 DX Genetic Analyzer) on whole peripheral blood and bone marrow samples. Chimerism analysis of granulocytic and CD3^+^ lymphocytic fractions was performed on peripheral blood samples.^13^

### Statistical Analysis and Definitions

Statistical analysis was performed with Jamovi Software (version 2.3.21). The Shapiro-Wilk test was used to check for data distribution. The chi-square test and Kruskal-Wallis test were used to compare categorical and continuous variables, respectively. PFS and OS were calculated from alloSCT to the first event (disease relapse or progression and/or death from any cause/last contact). Cumulative incidence of NRM, relapse, and acute and chronic GVHD (cGVHD) was calculated taking competitive risks into account. VOD definition and severity were assessed according to EBMT classification.^14^ Survival analyses were performed with the Kaplan-Meier method and log-rank test. Prognostic factors for NRM, PFS, and OS were identified using an univariable Cox proportional hazards model to assess hazard ratios (HRs). Multivariable Cox analysis was conducted for variables with *P* values <0.05 in the univariate analysis.

## RESULTS

### Population

A total of 66 consecutive patients undergoing alloSCT for myeloid neoplasms were analyzed. Fifty-eight patients had AML, whereas 8 had MDS, mainly with a high disease risk index (82%). Forty-eight patients (73%) received TF and 18 (27%) were treated with TTF as a conditioning regimen. Patient, disease, and transplant characteristics are summarized in Table 1. At alloSCT, the median age of the total population was 66 y (range, 46–76) and 56% of patients had an HCT-CI score of ≥3. Main comorbidities considered to calculate the HCT-CI score were: pulmonary dysfunction (n = 28; 42%), cardiac disease (n = 13; 20%), prior solid tumor (n = 11; 17%), and hepatic dysfunction (n = 11%; 17%).

**TABLE 1.** Main patient-, donor-, transplant features and outcome distributions among TF and TTF groups

Variable	Overall (N = 66)	TF (N = 48; 73%)	TTF (N = 18; 27%)	*P*
Patient age, y, median (range)	67 (46–76)	66 (46–76)	68 (49–73)	0.988
Sex; n (%)				0.059
Male	39/66 (59)	25/48 (52)	14/18 (78)	
Female	27/66 (41)	23/48 (48)	4/18 (22)	
HCT-CI, n (%)				0.842
0	12/66 (18)	8/48 (17)	4/18 (22)	
1–2	17/66 (26)	13/48 (27)	4/18 (22)	
≥3	37/66 (56)	27/48 (56)	10/18 (56)	
Disease, n (%)				0.488
AML	58/66 (88)	43/48 (90)	15/18 (83)	
MDS	8/66 (12)	5/48 (10)	3/18 (17)	
High DRI, n (%)	54/66 (82)	38/48 (79)	16/18 (89)	0.362
High-risk karyotype, n (%)	19/57 (33)	13/41 (32)	6/16 (37)	0.677
Disease status at HSCT, n (%)				**0.005**
Complete remission	52/66 (79)	42/48 (88)	10/18 (57)	
Marrow blasts >5%	14/66 (21)	6/48 (12)	8/18 (43)	
MRD status, n (%)				**0.005**
Positive	25/50 (50)	16/40 (40)	9/10 (90)	
Negative	25/50 (50)	24/40 (60)	1/10 (10)	
Previous lines of therapy, n (%)				**<0.001**
1	39/66 (59)	34/48 (71)	5/18 (28)	
2	19/66 (29)	13/48 (27)	6/18 (33)	
≥3	8/66 (12)	1/48 (2)	7/18 (39)	
Previous HSCT, n (%)	13/66 (20)	7/48 (15)	6/18 (33)	0.088
Donor, n (%)				
Matched related	10/66 (15)	9/48 (19)	1/18 (6)	0.183
Matched unrelated	23/66 (35)	16/48 (33)	7/18 (39)	0.673
Mismatched unrelated	11/66 (17)	10/48 (21)	1/18 (6)	0.138
Haploidentical	22/66 (33)	13/48 (27)	9/18 (50)	0.079
Graft source, n (%)				0.942
PBSC	64/66 (97)	46/48 (96)	18/18 (100)	
BMSC	2/66 (3)	2/48 (4)	0/18 (0)	
CD34^+^ ×10^6^ cells/kg, median (range)	6.2 (2.5–11.8)	6.3 (2.5–10.9)	6.1 (3.7–11.8)	0.846
CD3^+^ ×10^7^ cells/kg, median (range)	23.2 (1.7–64.0)	23.2 (1.7–60.0)	22.7 (5.8–64.0)	0.708
ATG, n (%)	38/66 (58)	30/48 (63)	8/18 (44)	0.186
PT-Cy, n (%)	26/66 (39)	16/48 (33)	10/18 (56)	0.100

AML, acute myeloid leukemia; ATG, anti-thymocyte globulin; BMSC, bone marrow stem cell; DRI, disease risk index; HCT-CI, hematopoietic cell transplantation-comorbidity index; HSCT, hematopoietic stem cell transplantation; MDS, myelodysplastic syndrome; MRD, minimal residual disease; PBSC, peripheral blood stem cell; PT-Cy, posttransplant cyclophosphamide; TF, treosulfan-fludarabine; TTF, thiotepa in combination with TF. Statistically significant *P* values (<0.05) are shown in bold.

AlloSCT was performed after first-line treatment in 37 patients (58%). Thirteen patients (20%) had already undergone previous alloSCT. Complete response status at transplant was achieved by 52 patients (79%); 25 of 50 patients (50%) were MRD-negative. Among non-CR and MRD-positive cases, 39 (61%) had active disease at alloSCT, whereas the remaining 26 (39%) were in CR MRD-negative status. AlloSCT was performed from matched (related or unrelated) donor in 35 cases (53%) and a haploidentical/mismatch donor in 31 cases (47%). Thirty-eight patients (58%) received ATG as GVHD prophylaxis, and PTCy was used in 26 cases (39%). Median CD34^+^ and CD3^+^ cells reinfused were 6.2 × 10^6^ cells/kg (range, 2.5–11.8) and 23.2 × 10^7^ cells/kg (range, 1.7–64.0), respectively. Engraftment, toxicities, and clinical outcomes are summarized in Table 2.

**TABLE 2.** Engraftment and clinical posttransplant outcomes among TF and TTF groups

Variable	Overall (N = 66)	TF (N = 48; 73%)	TTF (N = 18; 27%)	*P*
Engraftment, n (%)	63/65 (97)	45/47 (96)	18/18 (100)	0.278
Time to neutrophil engraftment, d, median (range)	15 (10–35)	15 (10–23)	15 (13–35)	0.069
Time to platelet engraftment, d, median (range)	15 (8–36)	14 (8–36)	16 (12–36)	**0.035**
Organ toxicity grade ≥3, n (%)	9/66 (14)	8/48 (17)	1/18 (6)	0.241
-Hepatic, n (%)	3/66 (5)	2/48 (4)	1/18 (6)	0.809
-Gastrointestinal, n (%)	2/66 (3)	2/48 (4)	0/18 (0)	0.379
-Cardiac, n (%)	2/66 (3)	1/48 (2)	1/18 (6)	0.464
-Renal, n (%)	2/66 (3)	1/48 (2)	1/18 (6)	0.464
Infection grade ≥3, n (%)	20/66 (30)	16/48 (33)	4/18 (22)	0.382
VOD, n (%)	4/66 (6)	3/48 (6)	1/18 (6)	0.916
100 d aGVHD grade ≥2 cumulative incidence, % (95% CI)	34 (22-45)	36 (22-50)	28 (7-49)	0.491
3-y cGVHD requiring treatment cumulative incidence, % (95% CI)	21 (11-32)	18 (3-32)	26 (2-47)	0.535
3-y NRM cumulative incidence, % (95% CI)	18 (9-28)	17 (5-25)	20 (0-36)	0.980
3-y relapse cumulative incidence, % (95% CI)	40 (28-53)	37 (26-54)	42 (20-56)	0.710
3-y OS, % (95% CI)	52 (41-68)	50 (36-70)	58 (37-93)	0.740
3-y PFS, % (95% CI)	46 (33-62)	44 (30-63)	50 (31-79)	0.990
Follow-up time, mo, median (range)	16 (1–96)	20 (1–96)	14 (1–39)	0.416

aGVHD, acute graft-versus-host disease; cGVHD, chronic GVHD; CI, confidence interval; NRM, nonrelapse mortality; OS, overall survival; PFS, progression-free survival; TF, treosulfan-fludarabine; TTF, thiotepa in combination with TF; VOD, veno-occlusive disease. Statistically significant *P* values (<0.05) are shown in bold.

TF and TTF groups had similar patient age, comorbidity burden, disease type and risk, donor type, and reinfused CD34^+^ and CD3^+^ graft content. Due to our strategy of conditioning assignment, the TTF group included more cytologically active disease (43% versus 12%, *P *= 0.005) and MRD-positive cases (90% versus 40%, *P *= 0.005) than the TF group. Globally, active disease (marrow blasts >5% or positive molecular marker) was significantly more frequent in the TTF group compared with TF (94% versus 48%, *P *< 0.001). Moreover, the proportion of heavily pretreated patients was higher in the TTF group compared with the TF group, including patients who received ≥2 lines of treatment before alloSCT (72% versus 30%, *P* < 0.001) or underwent a previous alloSCT (33% versus 15%, *P *= 0.088).

### Engraftment

One patient died from NRM before engraftment, whereas 2 patients failed engraftment due to disease persistence. All remaining 63 patients (97%) successfully engrafted. TF group had a significantly faster platelet engraftment compared with TTF (14 versus 16 d, *P *= 0.035), whereas no statistical difference emerged in neutrophil engraftment time. No statistical difference between the 2 groups was observed in the attainment of full-donor chimerism at first (*P *= 0.287) or at the third month (*P *= 0.648).

### Immune Reconstitution

With regard to immunological status before alloSCT, TF and TTF groups had similar levels of T cells CD3^+^ (804 versus 777/µL; *P *= 0.950), B cells CD19/CD20^+^ (38 versus 59/µL; *P *= 0.529), and natural killer (NK) cells CD3^−^CD16^+^CD56^+^ (160 versus 125/µL; *P *= 0.605). The T-cell reconstitution at 12 mo after alloSCT was slower in TTF in comparison with TF group, with CD3^+^ cell count of 1566/µL in TF group versus 757/µL in TTF group (*P *= 0.051) and CD3^+^CD8^+^ cell level of 1215/µL in TF group versus 485/µL in TTF group (*P *= 0.033). Moreover, B-cell recovery rates were similar in the TF and TTF groups at 6 mo (48% versus 33%; *P *= 0.525) and 12 mo (91% versus 83%; *P *= 0.568) after alloSCT. No other statistical differences in T-cell, B-cell, and NK-cell levels were observed between the 2 groups at 1, 3, 6, or 12 mo after alloSCT.

### Organ Toxicity and Infections

As reported in Table 2, severe organ toxicity (grade ≥3) within the first 100 d was observed in 9 patients (14%), involving hepatic (n = 3), followed by gastrointestinal (n = 2), cardiac (n = 2), and renal (n = 2). VOD has been considered separately and developed in 4 patients (6%) (3 severe, 1 very severe) and was successfully treated with defibrotide in 3 out of 4 cases. Severe (grade ≥3) infection rate within the first 100 d was 30%, with 16 cases of bacterial sepsis and 4 cases of invasive aspergillosis (3 pulmonary, 1 cerebral).

No significant difference was observed between TF and TTF in terms of severe organ toxicity (Table 2). Similarly, VOD (*P *= 0.916) and severe infections (*P *= 0.382) rates at 100 d after transplant were similar in the 2 groups.

### Graft-versus-host Disease

Acute GVHD (aGVHD) of any grade was observed in 28 patients (42%). Overall, 22 patients had a grade ≥2 aGVHD, with a cumulative incidence at 100 d after alloSCT of 34% (95% CI, 22-45). Twelve patients experienced cGVHD. Overall cumulative incidence of moderate and severe cGVHD was 17% (95% CI, 8-27) and 21% (95% CI, 11-32) at 1 and 3 y after alloSCT, respectively. As shown in Table 2, no statistical differences were noted in the cumulative incidence of aGVHD and cGVHD among TF and TTF groups.

### Relapse and Survival

**FIGURE 1.**
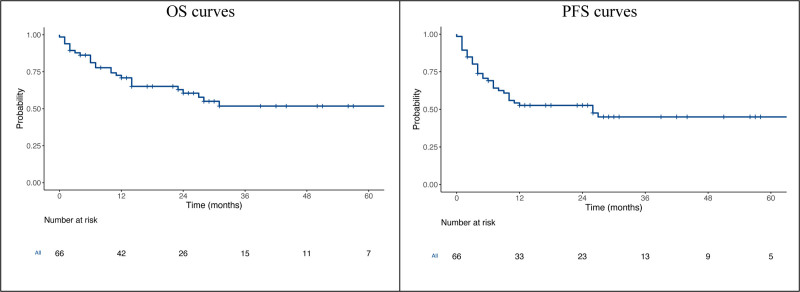
OS and PFS curves of the global population. OS, overall survival; PFS, progression-free survival.

**FIGURE 2.**
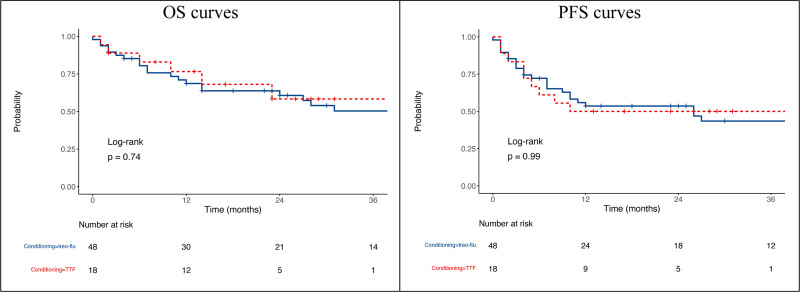
OS and PFS curves of TF (blue) and TTF (red). OS, overall survival; PFS, progression-free survival; TF, treosulfan-fludarabine; TTF, thiotepa in combination with TF.

Overall, median posttransplant follow-up was 16 mo (range, 1–96), with no significant difference between TF (20 mo; range, 1–96) and TTF (14 mo; range, 1–39) groups. Relapse or disease progression occurred in 24 patients (22 AML, 2 MDS) at a median time of 10 mo (range, 1–96) after alloSCT. Globally, the cumulative incidence of relapse was 33% (95% CI, 22-45) and 40% (95% CI, 28-53) at 1 and 3 y after alloSCT, respectively, with no statistical differences among TF and TTF groups. In univariable analysis (UVA), non-CR disease status at alloSCT (HR, 2.6; *P *= 0.043) and mismatched donor (HR, 2.5; *P *= 0.035) were associated with subsequent relapse. However, none of those variables were associated with disease relapse in multivariable analysis (MVA). At last-follow-up, 26 patients (39%) had died. The main causes of death were disease relapse (n = 15), infection (n = 7), GVHD (n = 3), and hemorrhage (n = 1). Cumulative incidences of NRM were 9% (95% CI, 3-17), 16% (95% CI, 8-26), and 18% (95% CI, 9-28) after 100 d, 1 y, and 3 y from alloSCT. Comparing groups, no difference in NRM cumulative incidence emerged after 100 d (8% in TF group, 11% in TTF group), 1 y (15% in TF group, 18% in TTF group), and 3 y (17% in TF group, 20% in TTF group; Table 2). In UVA, no variable was significantly associated with NRM. OS was 62% (95% CI, 52-74) and 52% (95% CI, 41-68) at 1 and 3 y after alloSCT, respectively (Figure 1), with no difference between conditioning groups (Figure 2). As reported in Table 3, treatment (≥2 previous lines) history (HR, 2.7; *P *= 0.029), non-CR disease status (HR, 3.1; *P *= 0.015), high-risk karyotype (HR, 2.4; *P *= 0.048), and mismatched donor (HR, 2.9; *P *= 0.017) adversely impacted OS in UVA. None of these variables was significantly associated with OS in the MVA. PFS was 51% (95% CI, 37-61) and 46% (95% CI, 33-62) at 1 and 3 y after alloSCT, respectively (Figure 1), with similar PFS curves retained by TF and TTF groups (Figure 2). Similarly to OS, non-CR disease status (HR, 2.8; *P *= 0.013), high-risk karyotype (HR, 2.3; *P *= 0.028), and mismatched donor (HR, 2.2; *P = 0.036*) adversely impacted PFS in UVA, whereas no variables were significantly associated with PFS in MVA. In all analyses, conditioning choice did not affect PFS, OS, and NRM.

**TABLE 3.** Univariable analysis in relation to PFS and OS

	PFS		OS	
Variables	HR (95% CI)	*P*	HR (95% CI)	*P*
Male sex	0.9 (0.4-2.2)	0.974	0.8 (0.4-2.0)	0.706
Age ≥65 y (at transplant)	0.8 (0.3-1.8)	0.550	1.3 (0.5-3.4)	0.669
HCT-CI >2	2.1 (0.9-5.0)	0.084	1.7 (0.7-4.1)	0.240
Previous lines ≥2	1.9 (0.9-3.9)	0.092	**2.7 (1.1-6.4**)	**0.029**
Previous alloSCT	2.0 (0.8-5.0)	0.151	2.1 (0.8-5.7)	0.164
High-risk karyotype	**2.3 (1.1-4.8**)	**0.028**	**2.4 (1.0-5.5**)	**0.048**
Non-CR disease status	**2.8 (1.2-6.4**)	**0.013**	**3.1 (1.2-7.6**)	**0.015**
MRD-positive disease status	1.7 (0.7-3.8)	0.225	1.2 (0.5-2.8)	0.687
Mismatched donor	**2.2 (1.1-4.7**)	**0.036**	**2.9 (1.2-7.0**)	**0.017**
ATG-based prophylaxis	0.6 (0.3-1.5)	0.296	0.6 (0.3-1.4)	0.229
TTF conditioning	0.7 (0.2-2.1)	0.556	0.6 (0.2-2.2)	0.487

alloSCT, allogeneic hematopoietic stem cell transplant; ATG, antithymocyte globulin; CI, confidence interval; CR, complete remission; HCT-CI, hematopoietic cell transplantation-comorbidity index; HR, hazard ratio; MRD, minimal residual disease; OS, overall survival; PFS, progression-free survival; TTF, thiotepa in combination with treosulfan-fludarabine. Statistically significant *P* values (<0.05) are shown in bold.

In the specific subgroup of patients undergoing second alloSCT (n = 13), 7 patients (54%) experienced relapse and 9 (69%) died (3 due to NRM). PFS was 28% (95% CI, 17-69) after 1 y and 17% (95% CI, 7-62) after 3 y; OS was 46% (95% CI, 16-67) after 1 y and 24% (95% CI, 5-73) after 3 y. No variable affected PFS nor OS in this specific setting, according to UVA.

## DISCUSSION

In the past decades, a significant increase in the age of transplant recipients has been reported in transplantation registries (https://www.gitmo.it/libro-gitmo-e-report-annuali/report-annuali-attivita-trapiantologica.html). However, the transplantation procedure in elderly patients has become more effective over time, with improvements in OS and cumulative incidence of relapse against a stability in NRM at 5 y, as reported by a large retrospective analysis conducted by the Italian Group of Bone Marrow Transplantation.^15^ Nowadays, the main alloSCT indication remains myeloid malignancies, with AML as the leading condition, accounting for 40% of procedures.^16^ Concerning haploidentical donor type use, an increase of 11.7% was noted in 2023 compared with 2022, probably due to the increased feasibility of alloSCT also in patients older than 65 y and with active disease at alloSCT.^16,17^ Treosulfan was the first drug approved for the reduced intensity preparative regimens of older or comorbid patients undergoing alloSCT.^3,4^ Nowadays, we face new challenges in the real-life application of TF regimen: first, even if the registrative study included only AML in remission, in clinical practice, patients with active disease might benefit from alloSCT; second, patients aged between 50 and 70 are very heterogeneous, and the younger of them could safely undergo myeloablative conditioning, if fit. Therefore, we planned to reserve TF for unfit patients—recognized by the criteria of the study by Beelen et al—among patients aged at least 65 y and those undergoing a second transplant. Moreover, we added thiotepa 5 mg/kg in patients with active disease to enhance antileukemic activity. Intensification of the TF conditioning has been experimented with using 2 different strategies: increasing the treosulfan dose to 12 or 14 g/m^2^ or adding a second alkylating agent, specifically thiotepa. Several studies on patients with AML and MDS revealed excellent efficacy and safety outcomes by using treosulfan 12 or 14 g/m^2^.^1,2^ Notably, a large retrospective study on 520 AML patients, including 188 cases with active at alloSCT, and mainly conditioned with TF 14 g/m^2^ (76%) showed 5-y NRM of 25%, 5 y OS 38%, and 5-y disease-free survival of 39%.^18^ However, all these studies were conducted in patients with a median age ranging between 50 and 57 y. More recently, a few retrospective studies reported data concerning the addition of a second alkylating agent (eg, thiotepa 5–10 mg/kg), even in AML and MDS patients older than 65 y.^9,19^ compared with the study by Beelen et al, our cohort included older patients (median age of 67 versus 60 ye), a higher amount of AML cases (88% versus 69%), fewer transplants from matched donors (53% versus 100%), and some transplants from haploidentical donors, which were excluded in the registrative study. Moreover, our study included patients with active disease (marrow blasts >5% or MRD positivity) and a second alloSCT. These differences in the features of our real-life population might explain the higher cumulative incidence of relapse (40% versus 26% at 3 y) and NRM (16 versus 12% at 2 y) in our study, as well as the lower OS rate (52% versus 67% at 3 y).^4^ However, we considered these results still of note out of randomized clinical trials, and supportive of the utility of alloSCT also in patients with active disease and/or the absence of a matched donor. Compared with the real-life results from an Italian Group of Bone Marrow Transplantation study, our population showed similar age (67 versus 64 y), a matched donor rate (53% versus 52%), and cytologically active disease at transplant (21% versus 29%). In terms of outcomes, our use of treosulfan-based regimens determined an inferior NRM (16% versus 29% at 2 y) and better OS (61% versus 53% at 2 y) rates, whereas relapse cumulative incidence was quite high in both studies (33% versus 27% at 2 y).^20^ Thiotepa addition in selected MDS and AML patients with active disease and low comorbidity burden resulted feasible outcome, ensuring sustained engraftment, limited toxicity, and efficient disease control. On the contrary, 3 patients receiving the TF regimen failed engraftment and resulted in disease persistence and death, suggesting a possible insufficient myeloablation activity by single alkylation use. Time to engraftment was similar between the 2 groups, and the small difference in platelet engraftment did not lead to higher hemorrhage rates in TTF compared with TF. In terms of immune reconstitution, no differences in peripheral B cells and NK cells were noted between the TTF and TF groups, whereas a slower recovery in CD3^+^CD8^+^ T cells was observed in TTF patients. In the absence of a significant difference in GVHD cumulative incidence at 1 y between groups, we hypothesize that a delay in T-cell recovery might be related to cytotoxic-induced thymic injury in heavily pretreated patients.^21^ Interestingly, even if the majority of TTF patients were highly pretreated and displayed active disease at alloSCT, no significant differences in terms of PFS and OS were noted, compared with TF. These results suggest that double alkylation might counterbalance the adverse risk given by the presence of active disease at alloSCT in selected cases, without adding toxicity or negatively affecting engraftment. Finally, our results emphasized that a second alloSCT with treosulfan-based conditioning is feasible and has high antileukemic efficacy in chemosensitive AML relapse after prior alloSCT. Given the limitations of the retrospective design and the relatively small number of patients, our results confirm the efficacy and tolerability of the TF regimen in older or comorbid AML/MDS patients in the real-world setting. The addition of thiotepa to TF in patients with active disease at alloSCT resulted feasible, well tolerated, and effective. Further larger and prospective studies are needed to confirm these data and better elucidate which older or comorbid AML/MDS patients could benefit from thiotepa addition.

## References

[1] Casper J Holowiecki J Trenschel R. Allogeneic hematopoietic SCT in patients with AML following treosulfan/fludarabine conditioning. Bone Marrow Transplant. 2012;47:1171–1177. 22158386 10.1038/bmt.2011.242

[2] Ruutu T Volin L Beelen DW. Reduced-toxicity conditioning with treosulfan and fludarabine in allogeneic hematopoietic stem cell transplantation for myelodysplastic syndromes: final results of an international prospective phase II trial. Haematologica. 2011;96:1344–1350. 21659356 10.3324/haematol.2011.043810 PMC3166105

[3] Beelen DW Trenschel R Stelljes M. Treosulfan or busulfan plus fludarabine as conditioning treatment before allogeneic haemopoietic stem cell transplantation for older patients with acute myeloid leukaemia or myelodysplastic syndrome (MC-FludT.14/L): a randomised, non-inferiority, phase 3. Lancet Haematol. 2020;7:e28–e39. 31606445 10.1016/S2352-3026(19)30157-7

[4] Beelen DW Stelljes M Reményi P. Treosulfan compared with reduced-intensity busulfan improves allogeneic hematopoietic cell transplantation outcomes of older acute myeloid leukemia and myelodysplastic syndrome patients: final analysis of a prospective randomized trial. Am J Hematol. 2022;97:1023–1034. 35617104 10.1002/ajh.26620

[5] Beelen DW Iacobelli S Koster L. Fludarabine-treosulfan versus fludarabine-melphalan or busulfan-cyclophosphamide conditioning in older AML or MDS patients—a clinical trial to registry data comparison. Bone Marrow Transplant. 2024;59:670–679. 38383713 10.1038/s41409-024-02241-2 PMC11073976

[6] Saraceni F Labopin M Raiola AM. Thiotepa-busulfan-fludarabine compared to treosulfan-based conditioning for haploidentical transplant with posttransplant cyclophosphamide in patients with acute myeloid leukemia in remission: a study from the acute leukemia working party of the EBMT. HemaSphere. 2023;7:e952. 37746158 10.1097/HS9.0000000000000952 PMC10513143

[7] Duléry R Bastos J Paviglianiti A. Thiotepa, busulfan, and fludarabine conditioning regimen in T cell-replete HLA-haploidentical hematopoietic stem cell transplantation. Biol Blood Marrow Transplant. 2019;25:1407–1415. 30871978 10.1016/j.bbmt.2019.02.025

[8] Saraceni F Beohou E Labopin M; Acute Leukemia Working Party (ALWP) of the European Society for Blood and Marrow Transplantation (EBMT). Thiotepa, busulfan and fludarabine compared to busulfan and cyclophosphamide as conditioning regimen for allogeneic stem cell transplant from matched siblings and unrelated donors for acute myeloid leukemia. Am J Hematol. 2018;93:1211–1219. 30033639 10.1002/ajh.25225

[9] Cassanello G Serpenti F Bagnoli F. Treosulfan, thiotepa and fludarabine conditioning regimen prior to first allogeneic stem cell transplantation in acute myeloid leukemia and high-risk myelodysplastic syndromes: a single center experience. Bone Marrow Transplant. 2023;58:1059–1061. 37355712 10.1038/s41409-023-02023-2

[10] Finke J Schmoor C Stelljes M. Thiotepa–fludarabine–treosulfan conditioning for 2nd allogeneic HCT from an alternative unrelated donor for patients with AML: a prospective multicenter phase II trial. Bone Marrow Transplant. 2022;57:1664–1670. 35982219 10.1038/s41409-022-01777-5 PMC9630110

[11] Sorror ML Maris MB Storb R. Hematopoietic cell transplantation (HCT)-specific comorbidity index: a new tool for risk assessment before allogeneic HCT. Blood. 2005;106:2912–2919. 15994282 10.1182/blood-2005-05-2004 PMC1895304

[12] Spyridonidis A Labopin M Savani BN. Redefining and measuring transplant conditioning intensity in current era: a study in acute myeloid leukemia patients. Bone Marrow Transplant. 2020;55:1114–1125. 31996792 10.1038/s41409-020-0803-y

[13] Béné MC Nebe T Bettelheim P. Immunophenotyping of acute leukemia and lymphoproliferative disorders: a consensus proposal of the European Leukemianet Work Package 10. Leukemia. 2011;25:567–574. 21252983 10.1038/leu.2010.312

[14] Mohty M Malard F Alaskar AS. Diagnosis and severity criteria for sinusoidal obstruction syndrome/veno-occlusive disease in adult patients: a refined classification from the European Society for Blood and Marrow Transplantation (EBMT). Bone Marrow Transplant. 2023;58:749–754. 37095231 10.1038/s41409-023-01992-8

[15] Malagola M Polverelli N Rubini V. GITMO registry study on allogeneic transplantation in patients aged ≥60 years from 2000 to 2017: improvements and criticisms. Transplant Cell Ther. 2022;28:96.e1–96.e11. 10.1016/j.jtct.2021.11.006 34818581

[16] Passweg JR Baldomero H Atlija M. The 2023 EBMT report on hematopoietic cell transplantation and cellular therapies. Increased use of allogeneic HCT for myeloid malignancies and of CAR-T at the expense of autologous HCT. Bone Marrow Transplant. 2025;60:519–528. 39939433 10.1038/s41409-025-02524-2 PMC11971038

[17] Maffini E Labopin M Kröger N. Allogeneic hematopoietic cell transplantation for older patients with AML with active disease. A study from the acute leukemia working party of the European Society for Blood and Marrow Transplantation (EBMT). Bone Marrow Transplant. 2024;59:983–990. 38555412 10.1038/s41409-024-02275-6

[18] Nagler A Labopin M Beelen D. Long-term outcome after a treosulfan-based conditioning regimen for patients with acute myeloid leukemia: a report from the acute leukemia working party of the european society for blood and marrow transplantation. Cancer. 2017;123:2671–2679. 28329410 10.1002/cncr.30646

[19] Piemontese S Lazzari L Ruggeri A. Allogeneic hematopoietic stem cell transplantation in patients older than 65 years with acute myeloid leukemia and myelodysplastic syndrome: a 15-year experience. Bone Marrow Transplant. 2022;57:678–680. 35124695 10.1038/s41409-022-01600-1

[20] Malagola M Polverelli N Martino M. Busulfan or treosulfan conditioning platform for allogeneic stem cell transplantation in patients aged >60 y with acute myeloid leukemia/myelodysplastic syndrome: a subanalysis of the GITMO alloeld study. Transplant Direct. 2023;9:e1451. 36845852 10.1097/TXD.0000000000001451 PMC9949804

[21] Velardi E Tsai JJ van den Brink MRM. T cell regeneration after immunological injury. Nat Rev Immunol. 2021;21:277–291. 33097917 10.1038/s41577-020-00457-z PMC7583557

